# Oral health in patients scheduled for hematopoietic stem cell transplantation in the Orastem study

**DOI:** 10.1371/journal.pone.0285615

**Published:** 2023-05-18

**Authors:** Kristina Skallsjö, Inger von Bültzingslöwen, Bengt Hasséus, Jan-Erik Johansson, Jenny Öhman, Judith E. Raber-Durlacher, Marie-Charlotte D. N. J. M. Huysmans, Alexa M. G. A. Laheij, Stephanie J. M. van Leeuwen, Allan J. Hovan, Karin Garming Legert, Hieu M. Nguyen, Philip J. Turk, Frederik R. Rozema, Nicole M. A. Blijlevens, Michael T. Brennan

**Affiliations:** 1 Department of Oral medicine and Pathology, Institute of Odontology, The Sahlgrenska Academy, University of Gothenburg, Gothenburg, Sweden; 2 Department of Oral Microbiology and Immunology, Institute of Odontology, The Sahlgrenska Academy, University of Gothenburg, Gothenburg, Sweden; 3 Department of Hematology and Coagulation, The Sahlgrenska Academy, University of Gothenburg, Gothenburg, Sweden; 4 Department of Oral Medicine, Academic Centre for Dentistry Amsterdam (ACTA), University of Amsterdam and VU University, Amsterdam, The Netherlands; 5 Department of Oral and Maxillofacial Surgery, Amsterdam UMC, Location University of Amsterdam, Amsterdam, The Netherlands; 6 Department of Dentistry, Radboud University Medical Center, Radboud Institute for Health Sciences, Nijmegen, The Netherlands; 7 Department of Preventive Dentistry, Academic Centre for Dentistry Amsterdam (ACTA), University of Amsterdam and VU University, Amsterdam, The Netherlands; 8 Oral Oncology and Dentistry, British Columbia Cancer, Vancouver, British Columbia, Canada; 9 Department of Dental Medicine, Karolinska Institutet, Huddinge, Sweden; 10 Center for Outcomes Research and Evaluation, Atrium Health, Charlotte, North Carolina, United States of America; 11 Department of Data Science, University of Mississippi Medical Center, Jackson, Mississippi, United States of America; 12 Department of Hematology, Radboud University Medical Center, Radboud Institute for Health Sciences, Nijmegen, The Netherlands; 13 Department of Oral Medicine/Oral & Maxillofacial Surgery, Atrium Health Carolinas Medical Center, Charlotte, North Carolina, United States of America; 14 Department of Otolaryngology/Head and Neck Surgery, Wake Forest University School of Medicine, Wake Forest, North Carolina, United States of America; University of Catania: Universita degli Studi di Catania, ITALY

## Abstract

Despite advances in transplant medicine, prevalence of complications after hematopoietic stem cell transplantation (HSCT) remains high. The impact of pre-HSCT oral health factors on the incidence and severity of complications post-HSCT is poorly understood. The aim of this prospective, observational study was to analyze oral health in patients planned for HSCT. Patients ≥18 years requiring HSCT were included from five sites between 2011–2018. General health, oral findings and patient-reported symptoms were registered in 272 patients. Oral symptoms around disease onset were reported by 43 patients (15.9%) and 153 patients (58.8%) reported oral complications during previous chemotherapy. One third of patients experienced oral symptoms at the oral examination before conditioning regimen and HSCT. In total, 124 (46.1%) patients had dental caries, 63 (29.0%) had ≥one tooth with deep periodontal pockets, 147 (75.0%) had ≥one tooth with bleeding on probing. Apical periodontitis was observed in almost 1/4 and partially impacted teeth in 17 (6.3%) patients. Oral mucosal lesions were observed in 84 patients (30.9%). A total of 45 (17.4%) of 259 patients had at least one acute issue to be managed prior to HSCT. In conclusion, oral symptoms and manifestations of oral disease were prevalent in patients planned for HSCT. The extent of oral and acute dental diseases calls for general oral screening of patients pre-HSCT.

## Introduction

Conditioning therapy followed by hematopoietic stem cell transplantation (HSCT) is an established and potentially curative treatment for many hematological malignancies, as well as other malignant and non-malignant disorders. The conditioning regimen consists of a combination of cytotoxic chemotherapeutic drugs, sometimes combined with total body irradiation (TBI), delivered before stem cell infusion. Despite advances in transplant medicine and supportive care, the prevalence of general and oral complications after HSCT remains high. Infections associated with neutropenia induced by cancer chemotherapy is a major cause of morbidity and mortality [1, 2]. Oral sequelae may jeopardize the general health of the patient during the post-transplantation phase and have a serious negative impact on a patient’s quality of life (QoL) [3, 4]. Oral complications may also have a long-term negative impact on treatment outcomes [3, 5, 6].

Oral health status may affect the incidence and severity of complications after HSCT [7, 8]. The oral cavity is a source of local and systemic infection due to compromised oral barrier functions and translocation of microorganisms colonizing oral mucosal surfaces in an immunocompromised host [9, 10]. Infections of dental origin may spread to become a systemic infection and lead to significant morbidity [11–15]. To reduce or prevent complications of oral origin, standard practice is to optimize oral hygiene before HSCT, provide necessary dental treatment and eliminating potential foci of infection. It is not clear which oral conditions warrant treatment and to what extent pre-HSCT dental treatment is needed [16, 17]. The possibility to perform dental treatment may be limited due to a patient’s medical condition, an urgency to start cancer treatment, extensive levels of dental diseases and, in some cases, a patient’s financial resources [14, 18, 19]. The risk for post-transplant complications arising from pre-HSCT dental treatment also needs to be considered since it may cause a delay in cancer treatment [14, 20]. Hence, careful procedures to ensure adequate oral assessment and treatment planning for patients scheduled for HSCT are essential.

Recent systematic reviews of oral complications associated with conditioning regimens and HSCT have confirmed the lack of knowledge regarding the overall oral health status of patients eligible for HSCT, the incidence and severity of complications of oral origin after HSCT [4, 21, 22], as well as how pre-HSCT oral health risk factors may influence such complications.

An international prospective observational multicenter study, the Orastem Study, was designed to establish the nature, incidence, temporal relationship, and burden of oral complications related to conditioning regimens, HSCT and a patient’s oral health. Additionally, the relationship between treatment related objective and subjective oral complications with negative clinical and economic outcomes and reduced QoL was assessed. The aim of this first Orastem substudy was to describe baseline oral health status, oral symptoms and factors associated with oral health in patients scheduled for HSCT.

## Material and methods

Patients were recruited at Sahlgrenska University Hospital, Gothenburg and Karolinska University Hospital Huddinge, Stockholm, Sweden; Atrium Health Carolinas Medical Center, Charlotte, NC, USA; BC Cancer, Vancouver, BC, Canada; Amsterdam UMC, University of Amsterdam, Amsterdam, The Netherlands and Radboud University Medical Center, Nijmegen, The Netherlands.

Patients ≥18 years old, scheduled for a conditioning regimen followed by autologous or allogeneic HSCT, were included in the study. Enrollment started at the first center in March 2011 and proceeded at intervals until May 2018. Initially, patients who were planned for full intensity or reduced intensity conditioning treatments were eligible for inclusion. From November 2016, patients planned for non-myeloablative conditioning were also eligible for inclusion. Exclusion criterion included patients unable to give consent. The Orastem Study protocol has been presented in detail [23]. Briefly, the whole study design includes five study phases starting with a baseline assessment before HSCT (Table 1). In this first substudy, data from the baseline assessment (phases I and IIa) are reported.

**Table 1:** Orastem study design.

Phase	Timepoint	Assessments	
Phase I	1–8 weeks before HSCT	Baseline pre-HSCT assessment *general health *patient-reported early oral problems related to disease requiring transplantation *oral health-related habits	
Phase II			
a	1–8 weeks before HSCT	Baseline pre-HSCT oral assessment *oral and dental status *patient-reported current oral problems	
b	Shortly before HSCT	Dental health assessment at HSCT *recent dental treatments (after oral examination before HSCT) *dental diseases left untreated *transplantation-related factors	
Phase III	Early post–transplantation, 3 days/week during hospitalization	Bedside post-HSCT assessment *oral examination *questionnaire on PRO at each visit	
Phase IV	Short-term follow-up	Supplementary checkups of oral problems after hospital discharge on demand of the dentist, patient’s physician, or patient	
Phase V	Long-term follow up	*Allogeneic HSCT*	*Autologous HSCT*
	100 days	*oral examination *PRO	*oral examination *PRO
	6 months	*oral examination *PRO	-
	12 months	*oral examination *PRO	*PRO

HSCT, hematopoietic stem cell transplantation

PRO, patient-reported outcome.

### Baseline pre-HSCT assessment

At the pre-HSCT assessment, scheduled 1–8 weeks before planned HSCT, age, gender, and anamnestic data on general health were collected. Data regarding current medication(s), medical diagnosis requiring HSCT, type of transplantation, previous radiotherapy to the head and neck, as well as previous and current bisphosphonate therapy were collected from medical records. Height and weight were recorded.

Patients were interviewed at baseline about experienced oral symptoms from around the time of onset of disease requiring HSCT and at previous chemotherapy treatments. Data regarding oral health-related habits, i.e., routine or not routine (only for acute problems or never) dental care and oral hygiene habits (frequency of tooth brushing and cleaning between teeth), tobacco and alcohol use (never, previous, current, and extent), were collected. Patients were considered as previous smokers or previous alcohol users if they had ceased smoking or ceased using alcohol, respectively, more than four weeks ago.

Baseline oral clinical assessment included a standard examination of oral hard and soft tissues, radiographic examination of teeth and surrounding tissues and registration of patient-reported current oral problems.

Number of teeth, dental implants, teeth with root canal filling, teeth with caries into dentin or pulp or pulpal exposure (due to caries, fracture, or wear) as well as number of symptomatic teeth, partially impacted teeth, asymptomatic and symptomatic apical periodontitis (AP) were recorded. Level of periodontal disease was documented by number of teeth with probing pocket depth (PD) >5mm and bleeding on probing (BoP), registered on four tooth surfaces when possible, considering risk of bleeding and infection susceptibility. Presence of supra- and subgingival calculus was registered by clinical examination, probing and/or radiographic examination. Oral hygiene was measured by no presence or presence of plaque visible to the naked eye, moderate or abundant plaque accumulation corresponding to grade 2 or 3 plaque accumulation, respectively, as described by Silness & Löe [24]. Oral hygiene in the present study was considered excellent if no teeth had visible plaque, good if 1–20% of teeth had visible plaque and intermediate or poor if 21–50% or >50% of teeth had visible plaque, respectively.

Oral mucosal lesions were described based on location, clinical appearance, and diagnosis. Microbiological samples and/or biopsies were obtained when clinically necessary. Oral mucositis was measured using the World Health Organization (WHO) toxicity scale (score 0–4) [25]. Stimulated whole salivary (SWS) flow rate was measured using paraffin chewing for 5 minutes and reported as mL/min.

Patient-reported current oral symptoms at the baseline pre-transplant oral assessment included symptoms from teeth, oral mucosa, and other oral problems. Subjective feeling of dry mouth (xerostomia) was graded with a numeric rating scale (NRS; 0–10).

### Ethical considerations and validation of data

Approval from the Ethical Review Boards at each study site was obtained. Sweden: Regional Ethical Review Board in Gothenburg (513–10, T939-16); Charlotte: Wake Forest School of Medicine Institutional Review Board (IRB00080071); Vancouver: BC Cancer Agency Research Ethics Board (BCCA REB # H11-02350, BCCA REB # H15-02350); Amsterdam and Nijmegen: Medical Ethical Research Committee, Amsterdam University Medical Center location AMC (NL52117.018.15), registered in the Dutch Trial Register (NL 5645). The approval granted in Amsterdam was validated by the IRB of Radboud UMC in Nijmegen. Written informed consent to participate in the study was provided by all patients. All data were registered and encrypted in a database using the computer software-program MedView [26]. All data were validated in several steps to identify systematic errors.

### Statistical analyses

Analyses for establishing oral health and oral symptoms at baseline are primarily descriptive in nature using frequency tables for categorical variables and summary statistics, such as median and range, for numerical variables. A comparison among enrollment sites with respect to stimulated salivary flow was made using the nonparametric Kruskal-Wallis test. Logistic regression models were utilized to investigate associations between medical diagnoses and patient-reported oral symptoms and complications pre-HSCT. In logistic regression models, the effects of interest were tested with likelihood ratio tests. Fisher´s exact test was used for analysis of the effect of oral hygiene on presence of pre-HSCT oral mucosal lesions. Association between SWS flow rate and patient reported grading of xerostomia was measured using the Kendall´s τ_B_ correlation. Because of the exploratory nature of this study, the reported *p*-values were not adjusted for multiplicity of tests, and a *p*-value less than or equal to a comparisonwise Type I error rate of *α* = 0.05 was considered statistically significant. Analyses were performed with R statistical software [27], version 4.0.3, and the SAS Enterprise Guide version 6.1 (SAS Institute Inc, Cary, North Carolina, USA).

A sample size was used to obtain an estimate of the prevalence of each individual oral complication within 0.06 of the true prevalence using a 95% confidence interval. Calculated sample sizes ranged from 62–254 patients [23]. Thus, the most conservative sample size of 254 patients was used. The total number of participants was higher, with the assumption that around 10% of inclusions would not proceed to HSCT.

## Results

**Fig 1:**
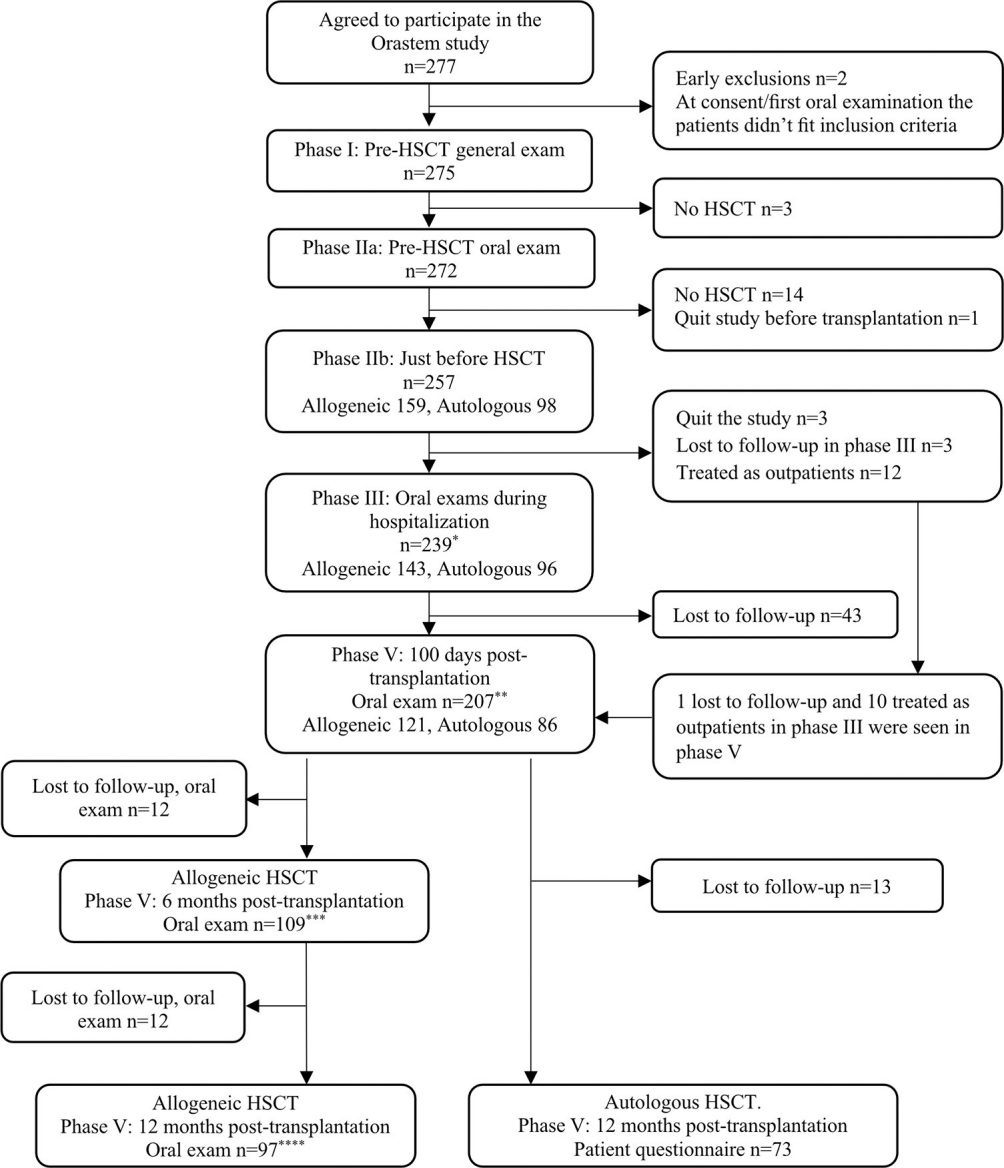
Orastem study flow chart showing number of patients in each phase throughout the study. *195 patients answered questionnaire, **161 patients answered questionnaire, ***92 patients answered questionnaire, ****96 patients answered questionnaire.

In total, 275 study participants went through the Orastem phase I pre-HSCT registration (median age 56.0 years, 42.2% females). A majority, 235 patients (85.5%), were assessed two weeks or more before planned HSCT, 10 patients (3.6%) were evaluated less than one week before. Of the 275 study participants, 272 also went through the phase IIa pre-HSCT oral examination. Altogether, 171 patients (62.9%) were scheduled for allogeneic and 101 (37.1%) for autologous transplantation. A flowchart of all Orastem Study phases is shown in Fig 1. Enrollment was done in accordance with clinical logistic procedures at each study center. In Vancouver, Amsterdam and Nijmegen, patients were enrolled consecutively during their enrollment periods. In Sweden and Charlotte, consecutive enrollment was not always possible for logistic reasons, as interruptions in inclusion during some time intervals occurred due to lack of personnel resources.

### General health

Most frequent medical diagnoses requiring transplantation were multiple myeloma (MM) n = 80, acute myeloid leukemia (AML) n = 67 and lymphoma n = 42. Baseline demographics and clinical characteristics are described in detail per center in Table 2. Since only five patients were included at Karolinska University Hospital in Stockholm, due to hospital reorganization, the two Swedish sites in Stockholm and Gothenburg are presented together (Sweden) since the patients at both sites are treated in accordance with the national treatment guidelines.

**Table 2:** Baseline demographics and clinical characteristics.

	All sites	Amst		Char		Nijm		Swed		Vanc	
No. of patients	275		43		57		81		38		56	
Gender												
Female	116 (42.2)		17 (39.5)		24 (42.1)		37 (45.7)		14 (36.8)		24 (42.9)	
Male	159 (57.8)		26 (60.5)		33 (57.9)		44 (54.3)		24 (63.2)		32 (57.1)	
Age, median; range	56.0;		60.0;		54.0;		57.0;		58.5;		55.0;	
	18–76		23–71		18–76		19–74		19–70		25–68	
Diagnosis												
AML	67 (24.4)		9 (20.9)		22 (38.6)		21 (25.9)		3 (7.9)		12 (21.4)	
ALL	17 (6.2)		1 (2.3)		6 (10.5)		4 (4.9)		2 (5.3)		4 (7.1)	
CLL	8 (2.9)		2 (4.7)		0 (0.0)		1 (1.2)		2 (5.3)		3 (5.4)	
CML	11 (4.0)		0 (0.0)		3 (5.3)		4 (4.9)		2 (5.3)		2 (3.6)	
Lymphoma	42 (15.3)		0 (0.0)		12 (21.1)		8 (9.9)		9 (23.7)		13 (23.2)	
MDS	18 (6.5)		1 (2.3)		3 (5.3)		8 (9.9)		2 (5.3)		4 (7.1)	
MM	80 (29.1)		29 (67.4)		5 (8.8)		26 (32.1)		9 (23.7)		11 (19.6)	
MPN, incl. myelofibrosis	15 (5.5)		1 (2.3)		3 (5.3)		4 (4.9)		3 (7.9)		4 (7.1)	
SAA	6 (2.2)		0 (0.0)		0 (0.0)		2 (2.5)		2 (5.3)		2 (3.6)	
Other diagnoses^a^	11 (4.0)		0 (0.0)		3 (5.3)		3 (3.7)		4 (10.5)		1 (1.8)	
BMI^b^, median; range	25.9;		25.5;		27.6;		25.0;		25.5;		27.7;	
	17.8–76.7		17.8–56.0		18.8–76.7		18.9–40.2		20.1–36.6		18.1–42.3	
Smoking^c^												
Never	136 (49.8)		17 (39.5)		33 (57.9)		35 (43.2)		18 (48.6)		33 (60.0)	
Previous	121 (44.3)		22 (51.2)		22 (38.6)		40 (49.4)		17 (45.9)		20 (36.4)	
Current	16 (5.9)		4 (9.3)		2 (3.5)		6 (7.4)		2 (5.4)		2 (3.6)	
Alcohol use^d^												
Never	65 (23.9)		5 (11.6)		17 (29.8)		12 (14.8)		4 (11.4)		27 (48.2)	
Previous	90 (33.1)		25 (58.1)		24 (42.1)		25 (30.9)		7 (20.0)		9 (16.1)	
Current	117 (43.0)		13 (30.2)		16 (28.1)		44 (54.3)		24 (68.6)		20 (35.7)	

*Note*: All entries are in numbers, n (%), except for age and BMI which are median (range).

ALL, acute lymphocytic leukemia; AML, acute myeloid leukemia; Amst, Amsterdam; BMI, Body Mass Index; Char, Charlotte; CLL, chronic lymphocytic leukemia; CML, chronic myeloid leukemia; MDS, myelodysplastic syndrome; MM, multiple myeloma; MPN, myeloproliferative neoplasms; Nijm, Nijmegen; SAA, severe aplastic anemia; Swed, Sweden; Vanc, Vancouver.

^a^ Other diagnoses: POEMS syndrome (n = 3); Prolymphocytic leukemia (n = 1); Chronic inflammatory demyelinating polyneuropathy (n = 1), Multiple sclerosis (n = 1), Hemophagocytic lymphohistiocytosis (n = 1), Paroxysmal nocturnal hemoglobinuria (n = 1), Hemoglobinopathy: Sickle-cell anemia (n = 3).

^b^ Missing data n = 19, ^c^ Missing data n = 2, ^d^ Missing data n = 3.

As shown, half of the patients had never smoked, almost half were previous smokers, while very few reported current smoking. A large majority of current or previous smokers, (83.9%) had smoked ≥5 years, and over half had smoked ≥10 cigarettes/day. Among the current smokers, five patients smoked a pipe or cigar. Only four patients reported use of smokeless tobacco. Of the 117 patients reporting current use of alcohol (Table 2), only 13 reported daily consumption.

Current medical conditions, beside the disease requiring HSCT, were reported in 145 (53.3%) subjects. The five most frequent medical conditions were hypertension (n = 44; 16.2%), diabetes mellitus (n = 24; 8.8%), anemia (n = 17; 6.3%), hypothyroidism (n = 12; 4.4%) and arthritis (n = 9; 3.3%). Almost 1/4 of patients (n = 65; 24.3%) reported prolonged bleeding, with the most frequent causes being thrombocytopenia due to disease or medical treatment (n = 43, 16.1%), anticoagulants (n = 12; 4.5%) and/or other bleeding disorders (n = 12; 4.5%).

A majority, 231 patients (87.5%), had one or more current medications. The five most frequent categories of drugs were antivirals (n = 112; 42.4%), antibiotics (n = 110; 41.7%), antiemetics (n = 63; 23.9%), antifungals (n = 58; 22.0%) and opioid analgesics (n = 57; 21.6%). Patients had an average of 4.3 categories (range 0–22) of medications. Almost one third of the patients (n = 83), most with MM, were under current or previous treatment with bisphosphonates (BP), 76 (91.6%) of these with intravenous BP, 7 (8.4%) with only oral BP. A few (n = 12) of the patients with intravenous BP, had also been treated with oral BP. Twelve patients with a mean age of 42.9 years (10 men, 2 women) had received prior radiotherapy to the head and neck area.

### Patient-reported early oral symptoms

#### Patient-reported oral symptoms at disease onset

Altogether, 43 patients (15.9%) experienced oral symptoms around the time of onset of disease requiring transplantation, and before medical treatment started. Overall, there were differences among diseases requiring HSCT with respect to the probability of these early symptoms (*p* = 0.001). Oral symptoms at disease onset were reported by 83.3% of patients with severe aplastic anemia (SAA), by 20–25% of patients with acute lymphoblastic leukemia (ALL), myelodysplastic syndrome (MDS) and acute myeloid leukemia (AML) and less frequently among patients with other diagnoses (Table 3A).

**Table 3:** Patient-reported oral symptoms a) around disease onset and b) from previous chemotherapy.

		Disease requiring HSCT									
	Total	AML	ALL	LYM	CLL	MDS	CML	MPN	SAA	MM	Other
***No*. *of patients***	***275***	***67***	***17***	***42***	***8***	***18***	***11***	***15***	***6***	***80***	***11***
**3a. Oral symptoms at disease onset**											
Missing data	4	0	1	2	1	0	0	0	0	0	0
Patients reporting oral symptoms at disease onset (%)	43/271 (15.9)	14/67 (20.9)	4/16 (25.0)	1/40 (2.5)	1/7 (14.3)	4/18 (22.2)	1/11 (9.1)	1/15 (6.7)	5/6 (83.3)	11/80 (13.8)	1/11 (9.1)
Type of oral symptom^*^, *n (%)*											
Gingival bleeding	16 (5.9)	4	1	0	1	2	1	0	3	4	0
Ulcer	14 (5.2)	6	1	0	0	2	0	1	1	2	1
Swollen gingiva	6 (2.2)	3	1	0	0	1	0	0	0	1	0
Bleeding from oral mucosa	5 (1.8)	2	0	0	0	1	0	0	2	0	0
Tooth ache	3 (1.1)	3	0	0	0	0	0	0	0	0	0
Infections	3 (1.1)	1	1	0	0	1	0	0	0	0	0
Other^a^	12 (4.4)	4	0	1	0	1	0	0	1	5	0
**3b. Oral symptoms from previous chemotherapy**											
Patients reporting never had chemotherapy	14	2	0	0	0	***3***	0	3	1	1	4
Missing data	1	0	0	1	0	0	0	0	0	0	0
Patients reporting oral symptoms from previous chemotherapy (%)	153/260 (58.8)	44/65 (67.7)	16/17 (94.1)	29/41 (70.7)	4/8 (50.0)	7/15 (46.7)	5/11 (45.5)	5/***12*** (41.7)	1/5 (20.0)	40/79 (50.6)	2/7 (28.6)
Type of oral symptom^*^, *n (%)*											
Taste changes	79 (30.4)	23	8	18	2	3	5	2	0	17	1
Dry mouth	71 (27.3)	14	8	20	1	3	4	2	0	19	0
Mucositis	32 (12.3)	12	4	6	2	1	0	0	1	6	0
Ulcerations	24 (9.2)	7	8	4	1	2	0	0	0	2	0
Tenderness	22 (8.5)	5	1	4	0	4	1	0	1	5	1
Pain	16 (6.1)	4	2	3	0	1	0	0	1	5	0
Blisters	15 (5.8)	3	4	1	0	2	0	0	0	4	1
Gingival/mucosal bleeding	12 (4.6)	3	0	3	0	1	1	0	1	2	0
Thrush fungal infections	10 (3.8)	1	1	4	1	0	0	0	0	1	2
Oral mucosal swelling	9 (3.5)	3	2	2	0	0	0	0	0	2	0
Burning sensation	8 (3.1)	1	1	4	0	0	0	0	0	2	0
Coated tongue	7 (2.7)	2	3	1	0	1	0	0	0	0	0
Halitosis	7 (2.7)	0	1	3	1	0	0	0	0	1	1
Other^b^	16 (6.2)	2	2	6	1	1	0	1	1	1	1

^a^ Other: Swollen jaw/cheek 2, Unfit denture 1, Other oral symptom, not specified 9.

^b^ Other: Herpes 4, Aphthae 3, Sandpaper feeling 3, Increased vomiting reflexes 2, Hoarseness 1, Myalgia 1, Bacterial infection 1, Allergic reaction 1.

* Multiple symptoms could be reported.

ALL, acute lymphocytic leukemia; AML, acute myeloid leukemia; CLL, chronic lymphocytic leukemia; CML, chronic myeloid leukemia; HSCT, hematopoietic stem cell transplantation; LYM, Lymphoma; MDS, myelodysplastic syndrome; MM, multiple myeloma; MPN, myeloproliferative neoplasms; SAA, severe aplastic anemia.

### Patient-reported oral symptoms from previous cancer chemotherapy

A vast majority of patients (n = 260, 94.9%) reported a medical history of previous treatment with cancer chemotherapy. Of these, over half (n = 153, 58.8%) reported experiences of oral symptoms in conjunction with the previous chemotherapy, with taste changes, dry mouth and oral mucositis being the most frequent (Table 3B). Transplant-requiring medical diagnosis had an impact on the probability of having an oral complication from earlier chemotherapy (*p* = 0.005) with ALL, AML, and lymphoma patients reporting the highest rates of oral complications (Table 3B).

### Oral health-related habits

The majority of patients reported routinely seeing a dentist or dental hygienist (Table 4), as opposed to never doing so (n = 9) or only for acute problems (n = 56). Regarding oral hygiene habits (Table 4), of the almost half who reported cleaning between teeth less than once per day, 62.8% (n = 81) did so less than once per week.

**Table 4:** Oral health-related habits in patients planned for hematopoietic stem cell transplantation (HSCT).

	All sites	Amst	Char	Nijm	Swed	Vanc
No. of patients	275	43	57	81	38	56
Routine dental care^a^						
Yes	208 (76.2)	37 (86.0)	30 (52.6)	70 (87.5)	32 (86.5)	39 (69.6)
No	65 (23.8)	6 (14.0)	27 (47.4)	10 (12.5)	5 (13.5)	17 (30.4)
Tooth-brushing^b^						
≥2/day	205 (76.5)	34 (79.1)	36 (63.2)	62 (79.5)	34 (91.9)	39 (73.6)
≤1 per day	63 (23.5)	9 (20.9)	21 (36.8)	16 (20.5)	3 (8.1)	14 (26.4)
Clean between teeth^c^						
≥1 per day	134 (51.0)	18 (41.9)	26 (46.4)	41 (55.4)	19 (51.4)	30 (56.6)
<1 per day	129 (49.0)	25 (58.1)	30 (53.6)	33 (44.6)	18 (48.6)	23 (43.4)

*Note*: Entries are number, n (%).

Amst, Amsterdam; Char, Charlotte; Nijm, Nijmegen; Swed, Sweden; Vanc, Vancouver.

^a^Missing data n = 2, ^b^Missing data n = 7, ^c^Missing data n = 12

### Dental findings

Dental findings, including reports on oral hygiene levels, are presented in detail in Table 5. Of the 272 patients who went through the baseline oral examination, 263 (97.0%) were dentate and eight (3.0%) were edentulous (1 missing answer). Almost half of patients (n = 124, 46.1%) had ≥1 tooth with caries into dentin or pulp, with a wide range of number of caries teeth (1–17) per patient, and 20 (7.4%) patients had ≥1 tooth with pulpal exposure due to caries, fracture, or wear. Almost one fourth had apical periodontitis (AP), with or without symptoms. Bleeding on probing (BoP) and PD >5 mm, were common (Table 5). The 17 (6.3%) patients with partially impacted teeth with communication to the oral cavity had an average of 1.3 (range 1–4) such teeth. Of the 22 partially impacted teeth, 21 (95.5%) were third molars and one was a canine.

**Table 5:** Dental health status at pre-hematopoietic stem cell transplantation (HSCT) baseline oral assessment.

	All sites	Amst	Char	Nijm	Swed	Vanc
**No. of patients**	272	43	56	81	38	54
**No. of teeth, median (range**)	26.0 (0–32)	26.0 (6–32)	27.0 (11–32)	26.0 (0–31)	28.5 (17–32)	26.5 (0–32)
**Dental caries**						
≥1 tooth with caries	124 (46.1)	16 (37.2)	40 (71.4)	50 (62.5)	11 (28.9)	7 (13.0)
No. of teeth with caries^a^	2.0 (1–17)	1.0 (1–8)	2.5 (1–17)	3.0 (1–12)	2.0 (1–9)	1.0 (1–6)
**Dental implants**	21 (7.8)	4 (9.3)	3 (5.4)	6 (10.1)	3 (7.9)	5 (9.3)
**Dental pulp and apical conditions**						
≥1 tooth with pulpal exposure	20 (7.4)	5 (11.6)	8 (14.5)	4 (5.1)	2 (5.3)	1 (1.9)
≥1 root-filled tooth	146 (53.9)	27 (62.8)	22 (39.3)	46 (57.5)	28 (73.7)	23 (42.6)
≥1 asymptomatic AP	55 (20.3)	5 (11.6)	14 (25.0)	19 (23.8)	12 (31.6)	5 (9.3)
≥1 symptomatic AP	7 (2.6)	1 (2.3)	2 (3.6)	3 (3.8)	1 (2.6)	0 (0.0)
≥1 symptomatic teeth, non-AP	6 (2.3)	0 (0.0)	2 (3.6)	0 (0.0)	4 (10.8)	0 (0.0)
**Impacted teeth**						
≥1 partially impacted tooth	17 (6.3)	5 (11.6)	5 (9.3)	1 (1.3)	4 (10.8)	2 (3.7)
**Periodontal disease** ^b^						
≥1 tooth with PD >5 mm.	63 (29.0)	12 (27.9)	12 (37.5)	25 (33.3)	6 (27.3)	8 (17.8)
No. of patients evaluated	217	43	32	75	22	45
No. of teeth with PD >5mm^a^	2.0 (1–16)	1.5 (1–8)	2.0 (1–9)	2.0 (1–16)	2.0 (1–14)	2.0 (1–15)
≥1 tooth with BOP	147 (75.0)	40 (93.0)	11 (47.8)	73 (97.3)	8 (72.7)	15 (34.1)
No. of patients evaluated	196	43	23	75	11	44
No. of teeth with BOP^a^	10.0 (1–30)	11.0 (2–29)	5.0 (1–15)	14.0 (1–30)	4.0 (1–26)	3.0 (1–22)
**Oral hygiene** ^b^						
No visible plaque	72 (30.0)	12 (30.8)	9 (20.9)	17 (22.4)	6 (17.6)	28 (58.3)
Plaque on 1–20% of teeth	91 (37.9)	11 (28.2)	17 (39.5)	30 (39.5)	21 (61.8)	12 (25.0)
Plaque on 21–50% of teeth	50 (20.8)	7 (17.9)	12 (27.9)	18 (23.7)	6 (17.6)	7 (14.6)
Plaque on more than 50% of teeth	27 (11.2)	9 (23.1)	5 (11.6)	11 (14.5)	1 (2.9)	1 (2.1)
No. of patients evaluated	240	39	43	76	34	48
Supragingival calculus	118 (50.6)	32 (78.0)	7 (23.3)	50 (64.1)	14 (42.4)	15 (29.4)
No. of teeth with supragingival calculus^a^	5 (1–27)	6 (1–27)	5 (3–8)	4 (1–10)	5 (3–10)	6 (3–20)
No. of patients evaluated	233	41	30	78	33	51
Subgingival calculus	61 (25.0)	7 (16.7)	20 (43.5)	24 (31.6)	8 (23.5)	2 (4.3)
No. of teeth with subgingival calculus^a^	4 (1–27)	2 (1–27)	4 (1–18)	3 (1–26)	7 (4–26)	4.5 (4–5)
No. of patients evaluated	244	42	46	76	34	46

*Note*: Entries are number, n (%) or median (range).

Missing answers: Number of teeth n = 1, Dental implants n = 2; Dental caries n = 3; Pulpal exposure n = 3, Root-filled teeth n = 1, Asymptomatic AP n = 1, Symptomatic AP n = 3, Symptomatic (not AP) n = 6; Partially impacted teeth n = 4.

Amst, Amsterdam; AP, apical periodontitis; BOP, Bleeding on probing; Char, Charlotte; Nijm, Nijmegen; PD, probing depth; Swed, Sweden; Vanc, Vancouver.

^a^Median number of teeth and range in patients with such dental conditions.

^b^Periodontal parameters were not measured for all patients. Number of patients evaluated regarding periodontal status as shown in table.

### Acute oral problems requiring treatment prior to HSCT

Dental and oral findings that constituted acute issues that needed to be addressed before HSCT included any of the following: symptoms of a sensitive tooth, tooth ache/symptomatic tooth with or without periapical lesion, tooth fracture, swelling from an infected tooth or erupting wisdom tooth, diffuse pain or myalgia and findings such as teeth with pulpal exposure. A total of 45 (17.4%) of 259 patients had at least one acute issue to be managed prior to HSCT.

### Oral mucosal findings

Clinical oral mucosal findings at baseline oral examination were observed in 84 (30.9%) patients. About one fourth of these patients presented with >1 lesion. The oral mucosal findings, presented based on diagnoses, results of diagnostic tests, clinical appearances and assessment of mucositis are shown in Table 6 with a categorization modified from Robledo-Sierra et al. [28]. There was not significant evidence that the proportion of patients with pre-HSCT oral mucosal lesions was different between levels of oral hygiene, good/excellent versus intermediate/poor, at this pre-HSCT examination (*p* = 0.456).

**Table 6:** Oral mucosal clinical findings at pre-hematopoietic stem cell transplantation (HSCT) baseline oral assessment.

Oral findings	n	n total
Mucosal infections		11
Fungal (2 culture-verified Candida albicans)	7	
Bacterial (2 culture verified)	2	
Viral (confirmed by PCR)	1	
Angular cheilitis	1	
Ulcers and Erosions		19
Ulceration NOS	6	
Mucositis (1 grade 1, 3 grade 2 according to WHO)	4	
Desquamation	4	
Erythema NOS	4	
Blister	1	
Denture related lesions		4
Denture related ulcer/pressure sore	2	
Denture stomatitis	1	
Denture hyperplasia	1	
Whitish lesions		10
Lichenoid lesions	5	
Leukoplakia	3	
Morsicatio	1	
Smokeless tobacco lesion	1	
Pigmentations		3
Submucosal hemorrhage, hematoma, petechiae		21
Mucosal overgrowth		15
Hyperplasia	8	
Hairy tongue	4	
Granulomatous tissue	2	
Leukemic infiltrate	1	
Other lesion/findings		29
Scalloping	11	
Edema NOS	5	
Food impaction (mucosal irritation)	1	
Other oral mucosal lesions, not specified	12	

*Note*: Entries are number, n, of oral mucosal lesions.

NOS, not otherwise specified; PCR, polymerase chain reaction; WHO, World Health Organization oral mucositis toxicity scale.

### Stimulated whole salivary flow

SWS flow rate was measured in 263 patients with an average amount of 1.3 mL/min (SD 0.8, median 1.1, range 0.02–4.6). There were differences between the study sites with respect to SWS flow rate (*p* < 0.001). Lowest values were recorded by patients in Charlotte, mean 0.9 mL/min, followed by Nijmegen 1.2 mL/min, Vancouver 1.4 mL/min, and Amsterdam 1.4 mL/min. Highest values at baseline were recorded in Sweden, mean 1.8 mL/min.

### Patient-reported oral symptoms at baseline pre-HSCT oral assessment

Almost one third, 81 patients (30.3%), reported one or more current oral symptoms at the baseline pre-transplant oral assessment (Table 7). The nature and distribution of any oral symptoms were followed up with specific questions regarding if the symptoms were from teeth, from oral mucosa, or if they were other oral symptoms.

**Table 7:** Patient-reported oral symptoms at baseline pre-hematopoietic stem cell transplantation (HSCT) assessment.

		Disease requiring HSCT									
	Total	AML	ALL	LYM	CLL	MDS	CML	MPN	SAA	MM	Other
**No. of patients**	**272**	**67**	**17**	**42**	**8**	**17**	**10**	**15**	**6**	**79**	**11**
Overall no. of patients (%) reporting any oral symptoms at baseline	81 (30.3)	15 (22.4)	4 (23.5)	10 (25.6)	1 (12.5)	4 (23.5)	5 (55.6)	5 (33.3)	3 (50.0)	30 (38.5)	4 (36.4)
Missing data	5	0	0	3	0	0	1	0	0	1	0
**Patients specifying symptoms involving teeth (%)**	45 (16.8)	5 (7.6)	4 (23.5)	8 (19.5)	1 (12.5)	2 (11.8)	3 (33.3)	3 (20.0)	2 (33.3)	15 (19.2)	2 (18.2)
Missing data	4	1	0	1	0	0	1	0	0	1	0
Type of symptom involving teeth^*^, *n*											
Sensitive teeth	16	2	2	3	0	1	0	0	1	6	1
Tooth or filling fracture	12	0	2	1	0	0	1	3	1	4	0
Food impaction	3	0	0	1	1	0	0	0	0	0	1
Oral swelling from infected tooth	1	1	0	0	0	0	0	0	0	0	0
Tooth ache	1	0	0	0	0	1	0	0	0	0	0
Other^a^	13	2	0	4	0	0	2	0	0	5	0
**Patients specifying symptoms involving oral mucosa (%)**	34 (13.0)	8 (12.3)	1 (5.9)	4 (10.8)	0 (0.0)	4 (23.5)	3 (33.3)	1 (6.7)	2 (33.3)	8 (10.4)	3 (27.3)
Missing data	10	2	0	5	0	0	1	0	0	2	0
Type of oral mucosal symptom^*^, *n*											
Gingival bleeding	7	2	0	1	0	1	0	0	2	1	0
Tenderness	7	2	0	0	0	2	2	0	0	1	0
Blisters	3	2	0	0	0	0	0	0	0	0	1
Oral mucosal edema/swelling	3	1	0	0	0	0	0	0	0	1	1
Aphthae	2	0	0	0	0	1	1	0	0	0	0
Burning sensation	2	0	0	0	0	0	0	0	0	2	0
Ulceration	2	0	0	0	0	0	1	0	0	1	0
Sand paper feeling	2	0	0	0	0	0	0	1	0	0	1
Other^b^	14	1	1	3	0	1	2	0	0	5	1
**Patients specifying other oral symptoms (%)**	72 (27.0)	12 (17.9)	5 (29.4)	13 (32.5)	2 (25.0)	1 (6.3)	5 (55.6)	4 (26.7)	0 (0.0)	27 (34.6)	3 (27.3)
Missing data	5	0	0	2	0	1	1	0	0	1	0
Type of other oral symptom^*^, *n*											
Dry mouth	46	6	4	7	1	0	1	3	0	22	2
Taste changes	28	7	0	5	1	1	3	2	0	8	1
Hoarseness	4	1	1	1	1	0	0	0	0	0	0
Other^c^	9	0	1	2	1	0	2	1	0	2	0

^a^Other: Pain/tenderness from teeth 2, strange feeling apically 1st right molar upper jaw 1, bite trauma 1, other symptom involving teeth not specified 9.

^b^Other: Coated tongue 1, hematoma 1, herpes intraoral 1, smarting sensation 1, pressure sore, tingling feeling 1, other oral symptom involving oral mucosa not specified 9.

^c^Other: Increased vomiting reflexes 1, diffuse pain 1, halitosis 1, limited mouth opening 1, other oral symptom, not specified 5.

* Multiple symptoms could be reported.

ALL, acute lymphocytic leukemia; AML, acute myeloid leukemia; CLL, chronic lymphocytic leukemia; CML, chronic myeloid leukemia; CT, chemotherapy; LYM, Lymphoma; MDS, myelodysplastic syndrome; MM, multiple myeloma; MPN, myeloproliferative neoplasms; SAA, severe aplastic anemia.

Of the 81 patients reporting current oral symptoms at baseline, over half (n = 45, 55.6%) reported symptoms from teeth, of which sensitive teeth (n = 16) and tooth or filling fracture (n = 12) were most frequent. Regarding other patient-reported dental symptoms at baseline, see Table 7.

Of the 81 patients who reported current oral symptoms, a number of patients had symptoms involving oral mucosa (n = 34, 42.0%), and other oral symptoms (n = 72, 88.9%), shown in detail in Table 7. Dry mouth (n = 46) was the most frequently reported oral symptom, followed by taste changes (n = 28). In the grading of a subjective feeling of dry mouth by NRS, which all patients were asked to do, more than twice as many (n = 104) reported oral dryness (>0) on the NRS, compared to the 46 who spontaneously reported dry mouth to the open question on oral symptoms. The 46 patients who had spontaneously reported dry mouth, had a mean NRS score of 4.5 (median 4). The patients that did not spontaneously report dry mouth to the open question but still reported NRS >0, had a lower mean NRS score of 3.0 (median 2). When analyzing the association between patients’ reported xerostomia (NRS>0) and SWS flow rate, NRS tended to decrease as SWS flow rate increased (Kendall´s τ_B_ correlation coefficient -0.16, *p* = 0.001).

## Discussion

Our study showed that oral findings and symptoms were prevalent in patients planned for HSCT. Certain medical diagnoses (SAA, AML, ALL and MDS) had a higher frequency of reported oral symptoms around disease onset compared to others. Certain medical diagnoses (ALL, lymphoma and AML) also had the highest frequency of reported oral complications during previous chemotherapy. At baseline assessment, almost half of the patients had dental caries, nearly one third had deep periodontal pockets and the same amount had oral mucosal lesions while approximately one fourth had apical periodontitis.

In total, 16% of patients reported that they had experienced oral symptoms around the time they became ill with the disease ultimately requiring transplantation. There are few comparable studies but, in a recently published study, Busjan et al. [29] reported that almost half of patients with newly diagnosed acute leukemia reported experiences of gum bleeding, swollen, painful, and/or sensitive gingiva during the last 12 months before diagnosis [29]. Watson et al. [30] reported that about 30% of patients with newly diagnosed acute leukemia, had clinical oral mucosal manifestations of their disease. The prevalence of patient-reported oral symptoms in the present study may be affected by measurement bias considering that patients were asked to recall the symptoms they had experienced around onset of disease. The lower overall prevalence of patient-reported oral symptoms in our study, compared to the studies by Busjan and Watson, may also be explained by the fact that more medical diagnoses requiring HSCT than acute leukemia were included. Patients with acute leukemia may frequently present with oral symptoms and manifestations, sometimes as initial manifestation of disease. In the present study, the most common patient-reported symptoms were bleeding from gingiva or oral mucosa, swollen gingiva and ulceration, which support findings reported by Busjan and Watson [29, 30].

Patients with SAA had the highest probability of experiencing oral symptoms around onset of disease, followed by acute leukemia and MDS. However, it needs to be recognized that the number of patients with SAA was small. Few large studies have been published on oral manifestations in patients with SAA. However, in accordance with our findings, Brennan et al [31] concluded that oral soft tissue manifestations in patients with aplastic anemia were frequent. We found that oral symptoms around disease onset were less frequent among patients with lymphoma, MPN, CML and MM. Reports of early onset oral symptoms and manifestations in patients with these medical diagnoses are scarce. Our study suggests that the relationship between medical diagnoses, especially SAA, acute leukemia and MDS, and the likelihood of oral problems around disease onset deserves further investigation.

Almost 60% of patients in our cohort recalled experiences of oral symptoms from previous chemotherapy. This is a high proportion, but less than recently published by Garcia-Chias et al. [32] who reported approximately 90% of patients receiving cancer chemotherapy for a solid tumor or hematological cancer experiencing oral side effects. In our study, taste changes and dry mouth were the most frequent oral problems from earlier chemotherapy. Similar problems but in higher frequency have been reported by others [32, 33]. The differences in prevalence may well be explained by the fact that our results are based on interviews with open questions on patients’ experiences of earlier treatments. Therefore, the answers could be subject to measurement bias. Patients with acute leukemia and lymphoma had the highest probability of experiencing oral symptoms from previous chemotherapy. There is limited knowledge on how oral side effects are related to type of malignancy and chemotherapy regimens [34, 35]. Patients at risk of side effects could gain from individualized information on expected complications and may have a larger need for supportive care after chemotherapy. Thus, our report on oral symptoms related to type of medical condition requiring transplantation and chemotherapy regimen adds important information that can be used in the clinical setting. Due to the small sample size of some of the medical diagnoses in our study, the results for each specific medical diagnosis need to be confirmed.

Dental diseases were prevalent in this cohort, which is in accordance with other studies in recent years [17, 18, 30, 36–38]. In our study, the overall incidence of periodontal diseases, measured clinically as PD >5 mm and BoP, was observed in almost 30% and 75% of patients, respectively. A limitation in the present study regarding data on periodontal disease is that not all patients could be assessed for periodontal status because blood cell counts did not permit invasive procedures. Therefore, it is possible that the level of periodontal disease at baseline could actually have been somewhat higher or lower than reported. To overcome these issues, one may use radiographic methods to estimate the level of periodontal disease (bone loss). However, this method also has limitations since it does not provide information on the actual level of gingival and periodontal inflammation.

One third of the patients had unsatisfactory (intermediate to poor) oral hygiene. Self-reported oral hygiene habits showed that the large majority of patients had regular tooth brushing habits, while cleaning between teeth on a regular basis was less frequent. Good oral hygiene have been reported to reduce the risk of complications such as infections and oral mucositis post-HSCT [39–42]. Therefore, providing professional dental cleaning and oral hygiene instruction for these patients is important. The pre-HSCT oral examination provides an excellent opportunity for the dental profession to provide individualized advice and instructions on oral hygiene practices. Dental disease and the need for dental treatment was seen in patients with a history of both regular and non-regular dental care. Therefore, attention to dental care needs is seen in all patients, regardless of dental care history.

Many patients reported being previous smokers at baseline, while few patients reported current smoking. Considering that more than 80% of “ever smokers” had smoked for many years, it cannot be ruled out that a number of patients may have quit smoking when diagnosed with the disease requiring transplantation. This may also be true regarding alcohol use.

The type of mucosal lesions found in one third of the patients at baseline were similar to findings by others [29, 30, 43]. However, most other publications largely concerned patients with acute leukemia. Since the baseline assessment was performed within weeks before planned transplantation, many lesions may be related to either the medical condition or treatment.

Dry mouth/xerostomia and taste changes were the most frequent oral problems reported by patients at baseline. The majority of patients had received previous chemotherapy at some timepoint, which may induce taste changes as well as xerostomia [35, 44, 45]. Interestingly, when patients graded their xerostomia on a numeric rating scale, more than twice as many patients reported xerostomia (NRS >0), compared to the ones that spontaneously reported xerostomia on an open question regarding oral problems. It is possible that the use of open questions, the heterogenicity of the medical diagnoses and the timing and range of prior chemotherapy had an influence on the prevalence levels of these symptoms. Our finding that xerostomia and taste changes are frequent oral symptoms in these patients is supported by earlier studies [32, 33, 35, 44], although questions remain on the impact of these problems on the overall well-being.

The average SWS in our study was slightly higher compared to what Uutela et al. [37] and Mauramo et al. [46] reported in similar cohorts. The SWS flow rate in our study was measured at baseline oral examination, while Uutela et al and Mauramo et al both measured SWS flow rate after conditioning therapy and immediately before HSCT. SWS flow rate differed between the sites, which could be explained by differences in medical diagnoses, national treatment protocols regarding number and types of prescribed medications, as well as other unknown factors. The association between SWS flow rate and xerostomia, measured by NRS, indicates that NRS may be a complementary tool to evaluate effects of SWS flow rate in these patients.

There is no standard of care for dental treatment in patients planned for HSCT and a recently published study indicated that chronic oral foci of infection did not increase infectious complications during intensive chemotherapy in patients who underwent autologous HSCT [38]. However, a decision analysis suggested that treatment prior to chemotherapy/HSCT may prevent the additional deaths of 18 out of every 10,000 patients and may reduce systemic infections by approximately one-third [11]. The fact that a notable part of the patients in our study presented with dental and mucosal diseases and findings weeks before HSCT calls for further studies on the impact of different oral diseases in these patient categories.

In summary, dental disease, oral findings and symptoms were common in this cohort of patients planned for HSCT. Almost one fifth presented with at least one acute dental disease. Based on the extent of oral symptoms and dental diseases found in our study, there is a need for more research regarding risk of complications after HSCT, how to prevent and reduce these complications and, in the long term, the impact of these conditions on general outcomes. The extent of acute dental issues calls for general oral screening of patients pre-HSCT.
