# Correction: Oral health in patients scheduled for hematopoietic stem cell transplantation in the Orastem study

**DOI:** 10.1371/journal.pone.0345853

**Published:** 2026-03-24

**Authors:** The PLOS One Staff

Tables 2–4 and 7 were uploaded incorrectly. Please see the correct Tables 2–4 and 7 here.

The publisher apologizes for the error.

**Table 2:** Baseline demographics and clinical characteristics.

	All sites	Amst	Char	Nijm	Swed	Vanc
No. of patients	275	43	57	81	38	56
Gender						
Female	116 (42.2)	17 (39.5)	24 (42.1)	37 (45.7)	14 (36.8)	24 (42.9)
Male	159 (57.8)	26 (60.5)	33 (57.9)	44 (54.3)	24 (63.2)	32 (57.1)
Age, median; range	56.0;	60.0;	54.0;	57.0;	58.5;	55.0;
	18–76	23–71	18–76	19–74	19–70	25–68
Diagnosis						
AML	67 (24.4)	9 (20.9)	22 (38.6)	21 (25.9)	3 (7.9)	12 (21.4)
ALL	17 (6.2)	1 (2.3)	6 (10.5)	4 (4.9)	2 (5.3)	4 (7.1)
CLL	8 (2.9)	2 (4.7)	0 (0.0)	1 (1.2)	2 (5.3)	3 (5.4)
CML	11 (4.0)	0 (0.0)	3 (5.3)	4 (4.9)	2 (5.3)	2 (3.6)
Lymphoma	42 (15.3)	0 (0.0)	12 (21.1)	8 (9.9)	9 (23.7)	13 (23.2)
MDS	18 (6.5)	1 (2.3)	3 (5.3)	8 (9.9)	2 (5.3)	4 (7.1)
MM	80 (29.1)	29 (67.4)	5 (8.8)	26 (32.1)	9 (23.7)	11 (19.6)
MPN, incl. myelofibrosis	15 (5.5)	1 (2.3)	3 (5.3)	4 (4.9)	3 (7.9)	4 (7.1)
SAA	6 (2.2)	0 (0.0)	0 (0.0)	2 (2.5)	2 (5.3)	2 (3.6)
Other diagnoses^a^	11 (4.0)	0 (0.0)	3 (5.3)	3 (3.7)	4 (10.5)	1 (1.8)
BMI^b^, median; range	25.9;	25.5;	27.6;	25.0;	25.5;	27.7;
	17.8–76.7	17.8–56.0	18.8–76.7	18.9–40.2	20.1–36.6	18.1–42.3
Smoking^c^						
Never	136 (49.8)	17 (39.5)	33 (57.9)	35 (43.2)	18 (48.6)	33 (60.0)
Previous	121 (44.3)	22 (51.2)	22 (38.6)	40 (49.4)	17 (45.9)	20 (36.4)
Current	16 (5.9)	4 (9.3)	2 (3.5)	6 (7.4)	2 (5.4)	2 (3.6)
Alcohol use^d^						
Never	65 (23.9)	5 (11.6)	17 (29.8)	12 (14.8)	4 (11.4)	27 (48.2)
Previous	90 (33.1)	25 (58.1)	24 (42.1)	25 (30.9)	7 (20.0)	9 (16.1)
Current	117 (43.0)	13 (30.2)	16 (28.1)	44 (54.3)	24 (68.6)	20 (35.7)

*Note*: All entries are in numbers, n (%), except for age and BMI which are median (range).

ALL, acute lymphocytic leukemia; AML, acute myeloid leukemia; Amst, Amsterdam; BMI, Body Mass Index; Char, Charlotte; CLL, chronic lymphocytic leukemia; CML, chronic myeloid leukemia; MDS, myelodysplastic syndrome; MM, multiple myeloma; MPN, myeloproliferative neoplasms; Nijm, Nijmegen; SAA, severe aplastic anemia; Swed, Sweden; Vanc, Vancouver.

^a^Other diagnoses: POEMS syndrome (n = 3); Prolymphocytic leukemia (n = 1); Chronic inflammatory demyelinating polyneuropathy (n = 1), Multiple sclerosis (n = 1), Hemophagocytic lymphohistiocytosis (n = 1), Paroxysmal nocturnal hemoglobinuria (n = 1), Hemoglobinopathy: Sickle-cell anemia (n = 3).

^b^Missing data n = 19, ^c^ Missing data n = 2, ^d^ Missing data n = 3.

**Table 3:** Patient-reported oral symptoms a) around disease onset and b) from previous chemotherapy.

		Disease requiring HSCT									
	Total	AML	ALL	LYM	CLL	MDS	CML	MPN	SAA	MM	Other
***No. of patients***	***275***	***67***	***17***	***42***	***8***	***18***	***11***	***15***	***6***	***80***	***11***
**3a. Oral symptoms at disease onset**											
Missing data	4	0	1	2	1	0	0	0	0	0	0
Patients reporting oral symptoms at disease onset (%)	43/271	14/67	4/16	1/40	1/7	4/18	1/11	1/15	5/6	11/80	1/11
	(15.9)	(20.9)	(25.0)	(2.5)	(14.3)	(22.2)	(9.1)	(6.7)	(83.3)	(13.8)	(9.1)
*Type of oral symptom* ^*^ *, n (%)*											
Gingival bleeding	16 (5.9)	4	1	0	1	2	1	0	3	4	0
Ulcer	14 (5.2)	6	1	0	0	2	0	1	1	2	1
Swollen gingiva	6 (2.2)	3	1	0	0	1	0	0	0	1	0
Bleeding from oral mucosa	5 (1.8)	2	0	0	0	1	0	0	2	0	0
Tooth ache	3 (1.1)	3	0	0	0	0	0	0	0	0	0
Infections	3 (1.1)	1	1	0	0	1	0	0	0	0	0
Other^a^	12 (4.4)	4	0	1	0	1	0	0	1	5	0
**3b. Oral symptoms from previous chemotherapy**											
Patients reporting never had chemotherapy	14	2	0	0	0	3	0	3	1	1	4
Missing data	1	0	0	1	0	0	0	0	0	0	0
Patients reporting oral symptoms from previous chemotherapy (%)	153/260	44/65	16/17	29/41	4/8	7/15	5/11	5/12	1/5	40/79	2/7
	(58.8)	(67.7)	(94.1)	(70.7)	(50.0)	(46.7)	(45.5)	(41.7)	(20.0)	(50.6)	(28.6)
*Type of oral symptom* ^*^ *, n (%)*											
Taste changes	79 (30.4)	23	8	18	2	3	5	2	0	17	1
Dry mouth	71 (27.3)	14	8	20	1	3	4	2	0	19	0
Mucositis	32 (12.3)	12	4	6	2	1	0	0	1	6	0
Ulcerations	24 (9.2)	7	8	4	1	2	0	0	0	2	0
Tenderness	22 (8.5)	5	1	4	0	4	1	0	1	5	1
Pain	16 (6.1)	4	2	3	0	1	0	0	1	5	0
Blisters	15 (5.8)	3	4	1	0	2	0	0	0	4	1
Gingival/mucosal bleeding	12 (4.6)	3	0	3	0	1	1	0	1	2	0
Thrush fungal infections	10 (3.8)	1	1	4	1	0	0	0	0	1	2
Oral mucosal swelling	9 (3.5)	3	2	2	0	0	0	0	0	2	0
Burning sensation	8 (3.1)	1	1	4	0	0	0	0	0	2	0
Coated tongue	7 (2.7)	2	3	1	0	1	0	0	0	0	0
Halitosis	7 (2.7)	0	1	3	1	0	0	0	0	1	1
Other^b^	16 (6.2)	2	2	6	1	1	0	1	1	1	1

^a^Other: Swollen jaw/cheek 2, Unfit denture 1, Other oral symptom, not specified 9.

^b^Other: Herpes 4, Aphthae 3, Sandpaper feeling 3, Increased vomiting reflexes 2, Hoarseness 1, Myalgia 1, Bacterial infection 1, Allergic reaction 1.

* Multiple symptoms could be reported.

ALL, acute lymphocytic leukemia; AML, acute myeloid leukemia; CLL, chronic lymphocytic leukemia; CML, chronic myeloid leukemia; HSCT, hematopoietic stem cell transplantation; LYM, Lymphoma; MDS, myelodysplastic syndrome; MM, multiple myeloma; MPN, myeloproliferative neoplasms; SAA, severe aplastic anemia.

**Table 4:** Oral health-related habits in patients planned for hematopoietic stem cell transplantation (HSCT).

All sites	Amst	Char	Nijm	Swed
275	43	57	81	38
208 (76.2)	37 (86.0)	30 (52.6)	70 (87.5)	32 (86.5)
65 (23.8)	6 (14.0)	27 (47.4)	10 (12.5)	5 (13.5)
205 (76.5)	34 (79.1)	36 (63.2)	62 (79.5)	34 (91.9)
63 (23.5)	9 (20.9)	21 (36.8)	16 (20.5)	3 (8.1)
134 (51.0)	18 (41.9)	26 (46.4)	41 (55.4)	19 (51.4)
129 (49.0)	25 (58.1)	30 (53.6)	33 (44.6)	18 (48.6)

*Note*: Entries are number, n (%).

Amst, Amsterdam; Char, Charlotte; Nijm, Nijmegen; Swed, Sweden; Vanc, Vancouver.

^a^Missing data n=2, ^b^Missing data n=7, ^c^Missing data n=12.

**Table 7:** Patient-reported oral symptoms at baseline pre-hematopoietic stem cell transplantation (HSCT) assessment.

		Disease requiring HSCT									
	Total	AML	ALL	LYM	CLL	MDS	CML	MPN	SAA	MM	Other
**No. of patients**	**272**	**67**	**17**	**42**	**8**	**17**	**10**	**15**	**6**	**79**	**11**
Overall no. of patients (%) reporting any oral symptoms at baseline	81	15	4	10	1	4	5	5	3	30	4
	(30.3)	(22.4)	(23.5)	(25.6)	(12.5)	(23.5)	(55.6)	(33.3)	(50.0)	(38.5)	(36.4)
Missing data	5	0	0	3	0	0	1	0	0	1	0
**Patients specifying symptoms involving teeth (%)**	45	5	4	8	1	2	3	3	2	15	2
	(16.8)	(7.6)	(23.5)	(19.5)	(12.5)	(11.8)	(33.3)	(20.0)	(33.3)	(19.2)	(18.2)
Missing data	4	1	0	1	0	0	1	0	0	1	0
*Type of symptom involving teeth* ^*^ *, n*											
Sensitive teeth	16	2	2	3	0	1	0	0	1	6	1
Tooth or filling fracture	12	0	2	1	0	0	1	3	1	4	0
Food impaction	3	0	0	1	1	0	0	0	0	0	1
Oral swelling from infected tooth	1	1	0	0	0	0	0	0	0	0	0
Tooth ache	1	0	0	0	0	1	0	0	0	0	0
Other^a^	13	2	0	4	0	0	2	0	0	5	0
**Patients specifying symptoms involving oral mucosa (%)**	34	8	1	4	0	4	3	1	2	8	3
	(13.0)	(12.3)	(5.9)	(10.8)	(0.0)	(23.5)	(33.3)	(6.7)	(33.3)	(10.4)	(27.3)
Missing data	10	2	0	5	0	0	1	0	0	2	0
*Type of oral mucosal symptom* ^*^ *, n*											
Gingival bleeding	7	2	0	1	0	1	0	0	2	1	0
Tenderness	7	2	0	0	0	2	2	0	0	1	0
Blisters	3	2	0	0	0	0	0	0	0	0	1
Oral mucosal edema/swelling	3	1	0	0	0	0	0	0	0	1	1
Aphthae	2	0	0	0	0	1	1	0	0	0	0
Burning sensation	2	0	0	0	0	0	0	0	0	2	0
Ulceration	2	0	0	0	0	0	1	0	0	1	0
Sand paper feeling	2	0	0	0	0	0	0	1	0	0	1
Other^b^	14	1	1	3	0	1	2	0	0	5	1
**Patients specifying other oral symptoms (%)**	72	12	5	13	2	1	5	4	0	27	3
	(27.0)	(17.9)	(29.4)	(32.5)	(25.0)	(6.3)	(55.6)	(26.7)	(0.0)	(34.6)	(27.3)
Missing data	5	0	0	2	0	1	1	0	0	1	0
*Type of other oral symptom* ^*^ *, n*											
Dry mouth	46	6	4	7	1	0	1	3	0	22	2
Taste changes	28	7	0	5	1	1	3	2	0	8	1
Hoarseness	4	1	1	1	1	0	0	0	0	0	0
Other^c^	9	0	1	2	1	0	2	1	0	2	0

^a^Other: Pain/tenderness from teeth 2, strange feeling apically 1st right molar upper jaw 1, bite trauma 1, other symptom involving teeth not specified 9.

^b^Other: Coated tongue 1, hematoma 1, herpes intraoral 1, smarting sensation 1, pressure sore, tingling feeling 1, other oral symptom involving oral mucosa not specified 9.

^c^Other: Increased vomiting reflexes 1, diffuse pain 1, halitosis 1, limited mouth opening 1, other oral symptom, not specified 5.

* Multiple symptoms could be reported.

ALL, acute lymphocytic leukemia; AML, acute myeloid leukemia; CLL, chronic lymphocytic leukemia; CML, chronic myeloid leukemia; CT, chemotherapy; LYM, Lymphoma; MDS, myelodysplastic syndrome; MM, multiple myeloma; MPN, myeloproliferative neoplasms; SAA, severe aplastic anemia.
